# An interactive nomogram to predict healthcare-associated infections in ICU patients: A multicenter study in GuiZhou Province, China

**DOI:** 10.1371/journal.pone.0219456

**Published:** 2019-07-15

**Authors:** Man Zhang, Huai Yang, Xia Mou, Lu Wang, Min He, Qunling Zhang, Kaiming Wu, Juan Cheng, Wenjuan Wu, Dan Li, Yan Xu, Jianqian Chao

**Affiliations:** 1 Key Laboratory of Environmental Medicine Engineering of Ministry of Education, Department of Medical Insurance, School of Public Health, Southeast University, Nanjing, China; 2 GuiZhou Healthcare Associated Infection Training Base, Center for Infectious Diseases, GuiZhou Provincial People’s Hospital, Guiyang, China; 3 Center for Infectious Diseases, Qiandongnan Prefecture People’s Hospital, Kaili, China; 4 Center for Infectious Diseases, Anshun City People's Hospital, Anshun, China; 5 Center for Infectious Diseases, Shuigang Hospital, Liupanshui, China; 6 Center for Infectious Diseases, Guizhou ShuiCheng Gold Mine Indestry Group general Hospital, Liupanshui, China; 7 Center for Infectious Diseases, Longli County People's Hospital, Qiannan Prefecture, China; 8 Key Laboratory of Environmental Medicine Engineering, Ministry of Education, School of Public Health, Southeast University, Nanjing, China; University of Maryland School of Medicine, UNITED STATES

## Abstract

**Objective:**

To develop and validate an interactive nomogram to predict healthcare-associated infections (HCAIs) in the intensive care unit (ICU).

**Methods:**

A multicenter retrospective study was conducted to review 2017 data from six hospitals in Guizhou Province, China. A total of 1,782 ICU inpatients were divided into either a training set (n = 1,189) or a validation set (n = 593). The patients’ demographic characteristics, basic clinical features from the previous admission, and their need for bacterial culture during the current admission were extracted from electronic medical records of the hospitals to predict HCAI. Univariate and multivariable analyses were used to identify independent risk factors of HCAI in the training set. The multivariable model’s performance was evaluated in both the training set and the validation set, and an interactive nomogram was constructed according to multivariable regression model. Moreover, the interactive nomogram was used to predict the possibility of a patient developing an HCAI based on their prior admission data. Finally, the clinical usefulness of the interactive nomogram was estimated by decision analysis using the entire dataset.

**Results:**

The nomogram model included factor development (local economic development levels), length of stay (LOS; days of hospital stay), fever (days of persistent fever), diabetes (history of diabetes), cancer (history of cancer) and culture (the need for bacterial culture). The model showed good calibration and discrimination in the training set [area under the curve (AUC), 0.871; 95% confidence interval (CI), 0.848–0.894] and in the validation set (AUC, 0.862; 95% CI, 0.829–0.895). The decision curve demonstrated the clinical usefulness of our interactive nomogram.

**Conclusions:**

The developed interactive nomogram is a simple and practical instrument for quantifying the individual risk of HCAI and promptly identifying high-risk patients.

## Introduction

Healthcare-associated infections (HCAIs) are the most common clinical complication and the second leading cause of death worldwide [1, 2]. HCAIs most commonly occur in the intensive care unit (ICU), with an estimated 17% to 51.4% of hospitalized ICU patients at risk for HCAIs [3, 4], increasing the number of days of hospitalization, medical expenses and mortality for ICU patients [5, 6]. Care for ICU patients by medical institutions is both time- and resource-consuming, focusing on all possible risk factors for HCAI in each patient admitted to the hospital [7]. If first-line healthcare workers can quickly screen high-risk HCAI patients based on their demographic and clinical characteristics from their last hospitalization, identify independent risk factors and implement specific proactive infection-control interventions during the current hospitalization, then they can not only help save medical resources and reduce the economic burden of disease but also reduce the incidence of HCAIs and improve patients’ quality of life [8]. Therefore, on the day that a patient is admitted to the ICU, predicting the highest risk of HCAI for the patient is very meaningful.

Previous studies have reported that independent factors affecting HCAIs include diabetes, chronic obstructive pulmonary disease and obesity [9, 10]. However, Teerawattanapong et al. did not find any links between these chronic diseases and HCAI, but instead observed that mechanical ventilation, antibiotic use, the presence of indwelling catheters and patient age were more likely contributing factors to the development of HCAIs [11]. In fact, the Centers for Disease Control and Prevention (CDC) guidelines for the identification of HCAI focus on patients requiring emergency surgery and indwelling catheters [12]. Piriyapatsom et al. reported that trauma patients were more likely to develop an HCAI [13], but Ferreira et al. and Chen et al. only found statistically significant associations among age, sex and HCAIs [3, 14]. Therefore, the independent risk factors for HCAI in ICU inpatients remain controversial. However, a systematic review by Gastmeier et al. showed that electronic surveillance was superior to traditional surveillance for ICU patients [15]. Electronic surveillance is not as time-consuming and costly as manual chart reviews (which typically involve examining chest computed tomography (CT), X-rays, procalcitonin levels, C-reactive protein (CRP) levels and routine blood test results), and the error rate for electronic surveillance is low, thus maximizing timely and effective HCAI prevention.

The nomogram model is a simple and practical tool for quantifying the individual risk for different diseases and for identifying high-risk patients, and has been verified in several studies [16, 17]. However, no effective nomogram is currently available to predict the risk of HCAI in hospitalized ICU patients. In this study, we developed a nomogram to identify patients at risk for HCAI during their current hospital admissions using data from their previous admissions [18]. Predictive models integrating information pertaining to certain factors can effectively stratify patients with different risk levels for HCAI. The objective of this work was to construct and evaluate a model using simple parameters to identify patients at high risk for acquiring HCAIs.

## Materials and methods

### Setting

Guizhou is a province of the People's Republic of China located in the southwestern part of the country, and the capital city is Guiyang. Guizhou is a relatively poor and economically undeveloped province that is rich in natural, cultural and environmental resources. The province has a humid subtropical climate with few seasonal changes.

### Study design

To obtain the maximum amount of information with minimal investment and construct a clinical nomogram model for HCAI prediction in ICUs in Guizhou Province, the cluster sampling method was adopted. We collected data and samples from Guizhou Provincial People’s Hospital, Anshun People’s Hospital, and Qiandongnan Prefecture People’s Hospital, Longli County People's Hospital, Shuigang Hospital and the Guizhou ShuiCheng Gold Mine Industry Group General Hospital. Data were obtained from the professional Xinglin monitoring system.

### Patients

The inclusion criteria were as follows: all ICU patients (with any previous admissions within 180 days) in the electronic medical records of the six hospitals above from January 1, 2017, to December 31, 2017, who were at least 18 years old and spent time in the ICU. The caret package [19] was used to extract training (n = 1,189) and validation (n = 593) samples from the identified cases based on a probability of 3:1 and a set.seed of 42 to ensure the consistency of the sampling results. Patients hospitalized for more than 180 days or fewer than 3 days and those who died within 48 h of admission to the ICU, were excluded from the study.

Retrospectively collected data for each included subject were retrieved from the Xinglin electronic medical records. All patient-specific features (such as chart number, name and ID) were reviewed and recoded. Age, sex, admission date, numbers of days of persistent fever (fever >38°C for more than two days), numbers of days in the ICU and hospital, disease history, surgical history, presence of invasive devices, antimicrobial treatment and discharge status were collected from prior admission records. The need for bacterial cultures at the time of admission was recorded when a patient was suspected of having any infection. For HCAI patients, we only recorded the first HCAI episode and retrieved the infection season, infection department, site of infection and microbiological results.

### Study endpoints

Our primary endpoint was the presence or absence of HCAI, which was a two-category variable. Other patient demographic characteristics and major clinical features were used as explanatory variables to predict the occurrence of endpoint events.

### Definition of HCAIs

The diagnostic criteria for HCAIs in this study were established according to the 2001 National Health and Family Planning Commission of the People’s Republic of China (NHFPC) Diagnostic Guidelines [20]. The main differences between the definition established by the NHFPC and that established by the US CDC/National Healthcare Safety Network (NHSN) are detailed in S1 Table. The differences between the two diagnostic guidelines are primarily related to the classification of lower respiratory tract (LRT) infections, the classification of infections at admission or HCAIs, and the definition of infections associated with devices. In this study, an HCAI was defined as an infection acquired within 48 h after admission, but cases with a clear infection latency according to the definition of the NHFPC were excluded. Community-acquired infection (CAI) was defined by a positive blood or bacteria culture obtained at the time of hospital admission or within 48 hours after hospital admission for patients who did not fit the criteria for an HCAI [21].

Because the NHFPC guideline does not cover device-associated infections, the device-related diagnostic criteria for infection in this study were based on a slightly revised definition of the CDC/NHSN monitoring definition criteria [22]. Relevant infections included ventilator-associated pneumonia (VAP), catheter-associated urinary tract infection (CAUTI) and central line-associated bloodstream infection (CLABSI), as described by Y. Chen [3]. In addition, related device infections contracted less than 48 hours after admission were not included in the study.

### HCAI surveillance program

The Xinglin system was developed based on the Diagnostic Criteria for Hospital Infection and the Code for Monitoring Hospital Infections. Based on the Hospital Information System (HIS), Laboratory Information System (LIS) and Remote Installation Service systems (RIS), the real-time monitoring and early-warning intervention system for HCAI was developed.

This system is a positive, web-based, real-time network established jointly by the General Hospital of the People's Liberation Army and Hangzhou Xinglin Information Technology Co., Ltd. in 2010. Real-time collection of HCAI-related information (including test results, clinical signs, medical records, and surgical records) was implemented to achieve infection tracking and real-time HCAI monitoring and to provide early warning and outbreak warning for patients from hospital admission to discharge. The data are automatically captured by the Xinglin system, which automatically screens suspicious cases of nosocomial infection for the full-time staff to judge. The system can save time when screening cases. On the one hand, this system can help full-time personnel handling infections in the hospital fully grasp the HCAI situation in the whole hospital. Furthermore, the system can allow these professionals to direct most of their energy towards the HCAIs in key departments. This approach not only improves the efficiency and accuracy of HCAI monitoring but also fundamentally changes the working mode of HCAI management professionals. At the same time, the system's interactive platform is used to transfer the HCAI cases identified by the full-time staff to clinicians for a definitive diagnosis. Therefore, six hospitals in Guizhou using the Xinglin system were selected as samples, which is very helpful for constructing predictive models. Because the information recorded by the Xinglin system is more objective and accurate, the information bias is minimized and information is more easily extracted.

The patients’ demographic characteristics, basic clinical features from the previous admission, and their need for bacterial culture during the current admission were extracted from the electronic medical records of the hospitals to predict HCAIs and construct an interactive nomogram. To maximize the accuracy of the model predictions, the interval between the previous hospitalization and the current hospitalization was not greater than 180 days, and if a patient had multiple hospital admissions, the data from the most recent hospital admission were used. All confirmed HCAI cases in this study met the NHFPC diagnostic criteria. To ensure high-quality internal consistency, patients were initially diagnosed by professionals with at least five years of infection control experience in various hospitals, and the final diagnosis was determined by senior hospital infection experts who had been employed for more than ten years; the external consistency of the diagnoses was good. Physicians who were involved in the diagnosis of nosocomial infections in the six hospitals received three months of standardized training at Guizhou Provincial People's Hospital. The six hospitals are part of a hospital consortium utilizing the same HCAI system to jointly diagnose difficult cases. The final enrollment of HCAI patients depended not only on the microbiological outcome but also on a patient's clinical symptoms, laboratory diagnosis, and imaging and pathology data. In addition, for the past five years, the hospital infection department has maintained a stable team, and the participating hospitals have implemented fixed management of their clinical departments.

### Statistical analysis

The Kolmogorov-Smirnov test was performed to assess the distribution equality of continuous parameters. Normally distributed data are presented as the means ± standard deviations, while skewed distribution data are presented as the medians and interquartile ranges. Independent *t*-tests and Mann-Whitney U tests were used to analyze differences in continuous variables between groups in the univariate analyses, while the chi-square test and the Fisher exact test were used for categorical variables.

A multivariable logistic regression analysis based on the results of the univariate analyses was performed to calculate the odds ratios (ORs) and 95% confidence intervals (CIs) of the independent variables.

The collinearity of the covariates was assessed, and model fitting was explored based on the Hosmer-Lemeshow goodness-of-fit test. The discriminatory ability of the model was assessed using the area under the receiver operating characteristic (ROC) curve. The best cutoff value for predicting the final model was selected based on the ROC cutoff value, and patients were stratified into a low- HCAI risk group and a high-HCAI risk group. A nomogram consisting of the independent risk factors was created to translate model parameter estimates into a visual scoring system to calculate the estimated probability of HCAI. More importantly, an interactive regression plot (regplot) visualization panel based on a multivariable regression model was employed to directly indicate the probability of HCAI occurrence per patient.

All statistical tests were performed using R statistical software version 3.5.1 (http://www.r-project.org). Statistical significance was assumed at P < 0.05.

### Ethics statement

The clinical ethics committees of the six hospitals (Guizhou Provincial People’s Hospital, Anshun People’s Hospital, Qiandongnan Prefecture People’s Hospital, Longli County People's Hospital, Shuigang Hospital and the Guizhou ShuiCheng Gold Mine Industry Group General Hospital) collectively reviewed the scientific and ethical rationale of the project and agreed to approve the project.

## Results

### Clinical characteristics

Table 1 presents the characteristics of the patients and the prevalence of HCAIs. Overall, 1,782 inpatients were enrolled in this study, including 410 (23.00%) with HCAIs and 1,372 (77.00%) without HCAIs. Most patients were men (1,191, 66.84%), and the average age of the inpatients was 60.06 ± 17.23 years. Among a total of 178 (9.99%) deceased patients, 120 (8.75%) did not have HCAIs, while 58 (14.15%) had HCAIs. Of the patients hospitalized for more than 36 days, 97 (5.44%) did not have HCAIs, and 139 (7.80%) had HCAIs. Compared with patients without HCAIs, those with HCAIs had an average increase of 5.35% in mortality and an average increase of 2.36% in the length of stay (LOS) over 36 days.

**Table 1:** Patient characteristics.

	Training set			Validation set		
Variables	Non-HCAI	HCAI	P	Non-HCAI	HCAI	P
	(n = 915)	(n = 274)		(n = 457)	(n = 136)	
Patients Age	59.76±17.63	62.50±16.13	0.054	58.13±17.24	63.60±15.66	0.002
Hospital Level			<0.001			<0.001
*general*	578 (63.17%)	107(39.05%)		274(59.96%)	49(36.03%)	
*large*	337 (36.83%)	167(60.95%)		183 40.04%)	87(63.97%)	
Local economic development levels			<0.001			0.059
*Undeveloped*	494 (53.99%)	109(39.78%)		237(51.86%)	58(42.65%)	
*developed*	421 (46.01%)	165(60.22%)		220(48.14%)	78(57.35%)	
Hospital Name			<0.001			<0.001
*People’s Hospital of ANSHUN*	194 (21.20%)	36(13.14%)		85(18.60%)	13(9.56%)	
*GuiZhou Provincial People’s Hospital*	337 (36.83%)	167(60.95%)		183(40.04%)	87(63.97%)	
*Qiandongnan Prefecture People’s Hospital*	165 (18.03%)	21(7.66%)		70(15.32%)	12(8.82%)	
*Longli County People's Hospital*	77 (8.42%)	10(3.65%)		41(8.97%)	8(5.88%)	
*Shuigang Hospital*	107 (11.69%)	33(12.04%)		54(11.82%)	15(11.03%)	
*SKZ*	35 (3.83%)	7(2.55%)		24(5.25%)	1(0.74%)	
Gender			0.194			<0.001
*female*	302 (33.01%)	79(28.83%)		178(38.95%)	32(23.53%)	
*male*	613 (66.99%)	195(71.17%)		279(61.05%)	104(76.47%)	
Admission Season			0.264			0.314
*summer*	209 (22.84%)	68(24.82%)		89(19.47%)	34(25.00%)	
*spring*	242 (26.45%)	76(27.74%)		129(28.23%)	29(21.32%)	
*autumn*	209 (22.84%)	70(25.55%)		113(24.73%)	36(26.47%)	
*winter*	255 (27.87%)	60(21.90%)		126(27.57%)	37(27.21%)	
Admission Clinic Department			0.016			<0.001
*internal*	203 (22.19%)	42(15.33%)		126(27.57%)	17(12.50%)	
*surgical*	341 (37.27%)	124(45.26%)		145(31.73%)	60(44.12%)	
*ICU*	371 (40.55%)	108(39.42%)		186(40.70%)	59(43.38%)	
Surgery			<0.001			0.006
*no*	594 (64.92%)	128(46.72%)		272(59.52%)	63(46.32%)	
*yes*	321 (35.08%)	146(53.28%)		185(40.48%)	73(53.68%)	
Need Bacterial Culture			<0.001			<0.001
*no*	305 (33.33%)	11(4.01%)		171(37.42%)	6(4.41%)	
*yes*	610 (66.67%)	263(95.99%)		286(62.58%)	130(95.59%)	
Diabetes			<0.001			<0.001
*no*	844 (92.24%)	202(73.72%)		423(92.56%)	96(70.59%)	
*yes*	71 (7.76%)	72(26.28%)		34(7.44%)	40(29.41%)	
Infection			<0.001			<0.001
*no*	614(67.10%)	152(55.47%)		317(69.37%)	71(52.21%)	
*yes*	301(32.90%)	122(44.53%)		140(30.63%)	65(47.79%)	
Cancer			<0.001			<0.001
*no*	859(93.88%)	215(78.47%)		430(94.09%)	96(70.59%)	
*yes*	56(6.12%)	59(21.53%)		27(5.91%)	40(29.41%)	
Hypertension			0.375			0.916
*no*	721(78.80%)	209(76.28%)		361(78.99%)	108(79.41%)	
*yes*	194(21.20%)	65(23.72%)		96(21.01%)	28(20.59%)	
Chronic Obstructive Pulmonary Disease			0.454			0.050
*no*	846(92.46%)	257(93.80%)		421(92.12%)	132(97.06%)	
*yes*	69(7.54%)	17(6.20%)		36(7.88%)	4(2.94%)	
Trauma			0.944			0.720
*no*	800(87.43%)	240(87.59%)		405(88.62%)	119(87.50%)	
*yes*	115(12.57%)	34(12.41%)		52(11.38%)	17(12.50%)	
Multiple Organ Failure			<0.001			0.088
*no*	886(96.83%)	252(91.97%)		442(96.72%)	127(93.38%)	
*yes*	29(3.17%)	22(8.03%)		15(3.28%)	9(6.62%)	
Days Of Hospital Stay			<0.001			<0.001
*≤6days*	332(36.28%)	15(5.47%)		153(33.48%)	2(1.47%)	
*7~14days*	330(36.07%)	48(17.52%)		151(33.04%)	36(26.47%)	
*15~36days*	194(21.20%)	121(44.16%)		115(25.16%)	49(36.03%)	
*36~64days*	45(4.92%)	42(15.33%)		19(4.16%)	27(19.85%)	
*>64days*	14(1.53%)	48(17.52%)		19(4.16%)	22(16.18%)	
Persistent Fever Days			<0.001			<0.001
*≤1days*	561(61.31%)	98(35.77%)		282(61.71%)	59(43.38%)	
*2~3days*	194(21.20%)	56(20.44%)		99(21.66%)	27(19.85%)	
*4~5days*	92(10.05%)	45(16.42%)		38(8.32%)	16(11.76%)	
*>5days*	68(7.43%)	75(27.37%)		38(8.32%)	34(25.00%)	
ICULOS			<0.001			<0.001
*≤6days*	561(61.31%)	58(21.17%)		269(58.86%)	22(16.18%)	
*7~10days*	160(17.49%)	40(14.60%)		69(15.10%)	25(18.38%)	
*11~36days*	156(17.05%)	121(44.16%)		97(21.23%)	54(39.71%)	
*>36days*	38(4.15%)	55(20.07%)		22(4.81%)	35(25.74%)	
Antibiotic Use Days			<0.001			<0.001
*≤3days*	274(29.95%)	11(4.01%)		127(27.79%)	6(4.41%)	
*4~6days*	220(24.04%)	22(8.03%)		113(24.73%)	12(8.82%)	
*7~12days*	237(25.90%)	56(20.44%)		116(25.38%)	29(21.32%)	
*13~17days*	61(6.67%)	35(12.77%)		47(10.28%)	20(14.71%)	
*18~37days*	100(10.93%)	95(34.67%)		43(9.41%)	43(31.62%)	
*>37days*	23(2.51%)	55(20.07%)		11(2.41%)	26(19.12%)	

People’s Hospital of ANSHUN City http://www.assrmyy.cn/

GuiZhou Provincial People’s Hospital in GUIYANG City http://www.5055.cn/

Qiandongnan Prefecture People’s Hospital Qiandongnan minority regions http://www.qdnzrmyy.net/

Longli County People's Hospital in Qiannan minority regions

SHUIGANG HOSPITAL in LIUPANSHUI City http://www.sgsgzyy.com/

SCZ, Guizhou ShuiCheng Gold Mine Indestry Group general Hospital in LIUPANSHUI City https://yyk.99.com.cn/shuicheng/87800/jianjie.html

HCAI, healthcare associated infections. Hospital Level (in 2017, more than 1,500 beds in the hospital were defined as large, and less than 1,500 beds were defined as general).ICULOS, ICU length of stay.

### Risk factors for HCAI in ICU inpatients

Table 2 shows the independent risk factors for ICU inpatients. With results reported as ORs (95% CI), the following factors were shown to be independently associated with HCAI: local economic development levels [in 2017, a real gross domestic product (GDP) per capita greater than 35,000 yuan was defined as developed, while a GDP less than 35,000 was defined as underdeveloped] [23], culture (the need for bacterial culture), diabetes (history of diabetes), cancer (history of cancer), LOS (days of hospitalization) and fever (days of persistent fever).

**Table 2:** Risk factors for ICU inpatients in the training set.

Variable	group	Univariate		Multivariable		
		OR (95% CI)	P	β^a^	OR (95% CI)	P
Intercept	-	-	-	-5.60		
Local economic development levels	*undeveloped*	1				
	*developed*	2.89(1.94~4.31)	<0.001	1.00	2.71(1.89~3.87)	<0.001
The need for bacterial culture	*no*	1				
	*yes*	6.84(3.28~14.29)	<0.001	2.11	8.28(4.15~16.54)	<0.001
Diabetes	*no*	1				
	*yes*	3.16(1.92~5.19)	<0.001	1.01	2.74(1.74~4.31)	<0.001
Cancer	*no*	1				
	*yes*	3.51(2.05~6.01)	<0.001	1.17	3.23(1.95~5.35)	<0.001
Days Of Hospital Stay						
	*≤6 days*	1				
	*7~14 days*	1.53(0.71~3.3)	0.27	0.75	2.12(1.12~3.99)	0.020
	*15~36 days*	3.66(1.61~8.32)	<0.001	2.09	8.12(4.45~14.83)	<0.001
	*36~64 days*	3.31(1.22~8.97)	0.02	2.29	9.84(4.73~20.46)	<0.001
	*>64 days*	20.01(6.17~64.86)	<0.001	4.08	59.01(24.8~140.36)	<0.001
Persistent Fever Days						
	*≤1 day*	1				
	*2~3 days*	1.08(0.67~1.75)	0.75	0.05	1.05(0.67~1.66)	0.833
	*4~5 days*	1.51(0.87~2.62)	0.15	0.46	1.59(0.94~2.68)	0.085
	*>5 days*	2.78(1.63~4.74)	<0.001	1.11	3.04(1.86~4.95)	<0.001
Hospital Level	*general*	1				
	*large*	1(0.66~1.52)	0.99			
Patients Age		1(0.99~1.02)	0.48			
Gender	*female*	1.08(0.73~1.59)	0.71			
	*male*					
Admission Season						
	*summer*	1				
	*spring*	1.38(0.85~2.26)	0.20			
	*autumn*	0.95(0.57~1.57)	0.83			
	*winter*	0.65(0.39~1.07)	0.09			
Admission Clinical Department						
	*internal*	1				
	*surgical*	1.61(0.93~2.79)	0.09			
	*ICU*	1.2(0.69~2.1)	0.51			
Surgery	*no*	1				
	*yes*	1.32(0.89~1.96)	0.17			
Infection	*no*	1				
	*yes*	0.8(0.54~1.2)	0.28			
Hypertension	*no*	1				
	*yes*	0.62(0.39~0.99)	0.05			
Chronic Obstructive Pulmonary Disease	*no*	1				
	*yes*	0.9(0.4~1.99)	0.79			
Trauma	*no*	1				
	*yes*	0.86(0.5~1.5)	0.60			
Multiple Organ Failure	*no*	1				
	*yes*	1.78(0.8~3.95)	0.16			
Antibiotic Use Days	*≤3 days*	1				
	*4~6 days*	1.48(0.65~3.37)	0.35			
	*7~12 days*	1.97(0.86~4.49)	0.11			
	*13~17 days*	2.75(1.09~6.96)	0.03			
	*18~37 days*	3.93(1.62~9.53)	<0.001			
	*>37 days*	6.07(1.93~19.11)	<0.001			

Model: Logit (HCAI) = -5.60+2.11*(Culture = 1/0)+1.01*(Diabetes = 1/0)+1.17*(Cancer = 1/0) +1.00* (Economic = 1/0)+0.75*(LOS = 7~14days)+2.09*(LOS = 15~36days)+2.29*(LOS = 36~64days)+4.08*(LOS = >64days)+0.05*(Fever = 2~3days) + 0.46* (Fever = 4~5days) + 1.11 * (Fever = >5days)

OR, odds ratio; CI, confidence interval; P ≤ 0.05 was considered to indicate statistical significance

COPD, Chronic Obstructive Pulmonary Disease; HCAI, healthcare associated infections

Hospital Level (in 2017, more than 1,500 beds in the hospital were defined as large, and less than 1,500 beds were defined as general); Local economic development levels (In 2017, real gross domestic product per capita (yuan) is defined as developed, less than 35,000 is defined as underdeveloped)

^a^ Unstandardized β coefficients were calculated from the multivariate logistic regression model.

### Assessment of model performance

**Fig 1:**
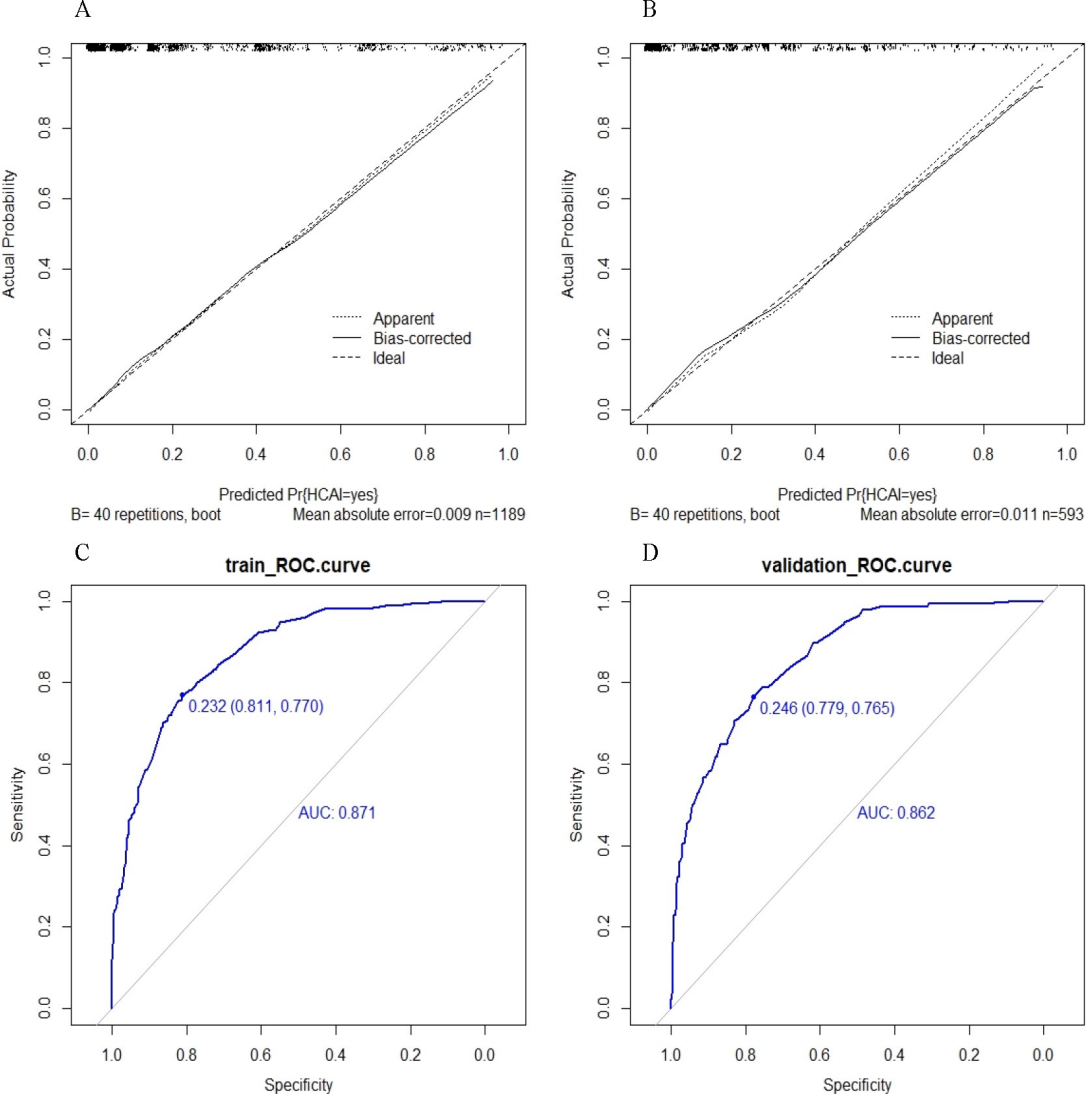
Goodness of fit of the predicted risk and actual risk of healthcare associated infections. A Calibration curves of the multiple regression model in the training set. B Calibration curves of the multiple regression model in the validation set. C The ROC curves of the in the multiple regression model training sets. D the ROC curves of the in the multiple regression model validation sets. Calibration curves depict the calibration of the multiple regression model in terms of agreement between the predicted risk of HCAI and observed HCAI outcomes. The 45-degree long dotted line represents a perfect prediction, and the solid line represent the predictive performance of the multiple regression model. The closer the long dotted line fit is to the ideal line, the better the predictive accuracy of the model is. ROC curves depict discrimination capability of nomogram model. The larger the area of the AUC, the higher the prediction accuracy of the model. The closer the predicted value is to the actual value. HCAI, healthcare associated infections.

The evaluation results of the multiple regression model performance is shown in Fig 1. Regarding outlier diagnoses, the model result covariate was approximately ± 1, suggesting the absence of outliers (S1 Fig). The variance inflation factor (VIF) values of the six covariates ranged from 1.10 to 3.11, indicating no collinearity in the model. A shows a calibration curve for the multiple regression model in the training set. The calibration curve and Hosmer-Lemeshow test (P = 0.366) performed well in the training set. An area under the curve (AUC) of 0.871 (95% CI, 0.8484–0.8936) indicated good differentiation of the model in the training set (C). The calibration map of the HCAI multiple regression model was confirmed using the validation set (B). The Hosmer-Lemeshow test showed that the P value was not significant at 0.591, and the AUC of the validation group (D) was 0.862 (95% CI, 0.8292–0.895). Therefore, our conclusion is that the multiple regression model performed well in both the training and validation sets.

### Clinical utility of the model

**Fig 2:**
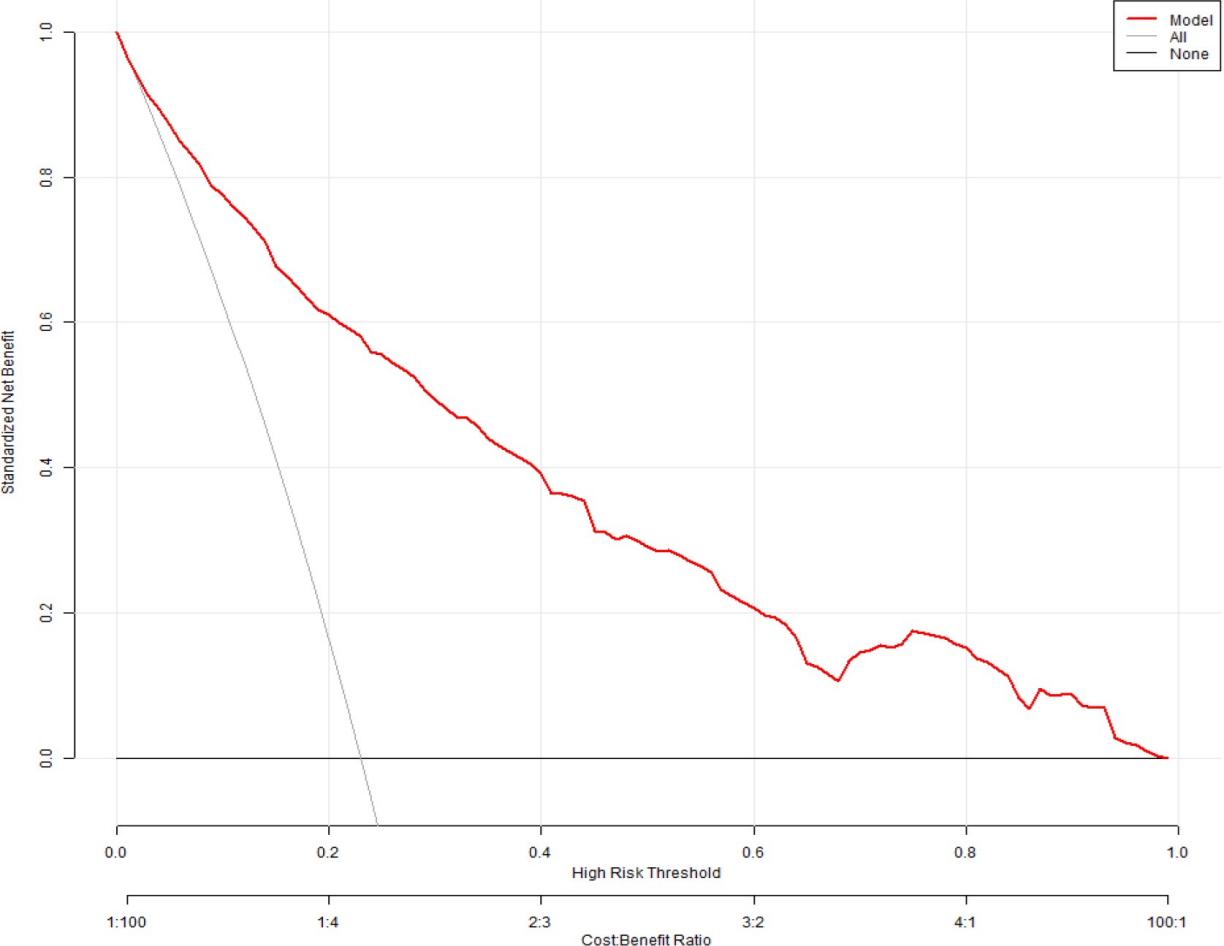
Decision Curve Analysis for prediction healthcare-associated infections multiple regression model. The y-axis represents the net benefit. The red line represents the multiple regression model. The dotted line represents the hypothesis that all patients had HCAI. Located above the High risk threshold line, and the black line parallel to the X axis represents “No HCAI”. The x-axis represents the threshold probability. The threshold probability is where the expected benefit of treatment is equal to the expected benefit of avoiding treatment. For example, if the possibility of HCAI involvement of a patient is over the threshold probability, then a treatment strategy for high risk patient should be adopted. The decision curves in the validation set showed that if the threshold probability is between 0 and 0.97, then using the multiple regression model to predict HCAI adds more benefit than focus on either all or no patients.

Fig 2 presents a decision curve analysis (DCA) curve for predicting the HCAI multiple regression model. In the DCA curve, when a patient's HCAI threshold probability was in the range of 0–0.97, the multiple regression model added a greater net benefit than the “treat all” or “treat none” strategies.

### Nomogram construction

**Fig 3:**
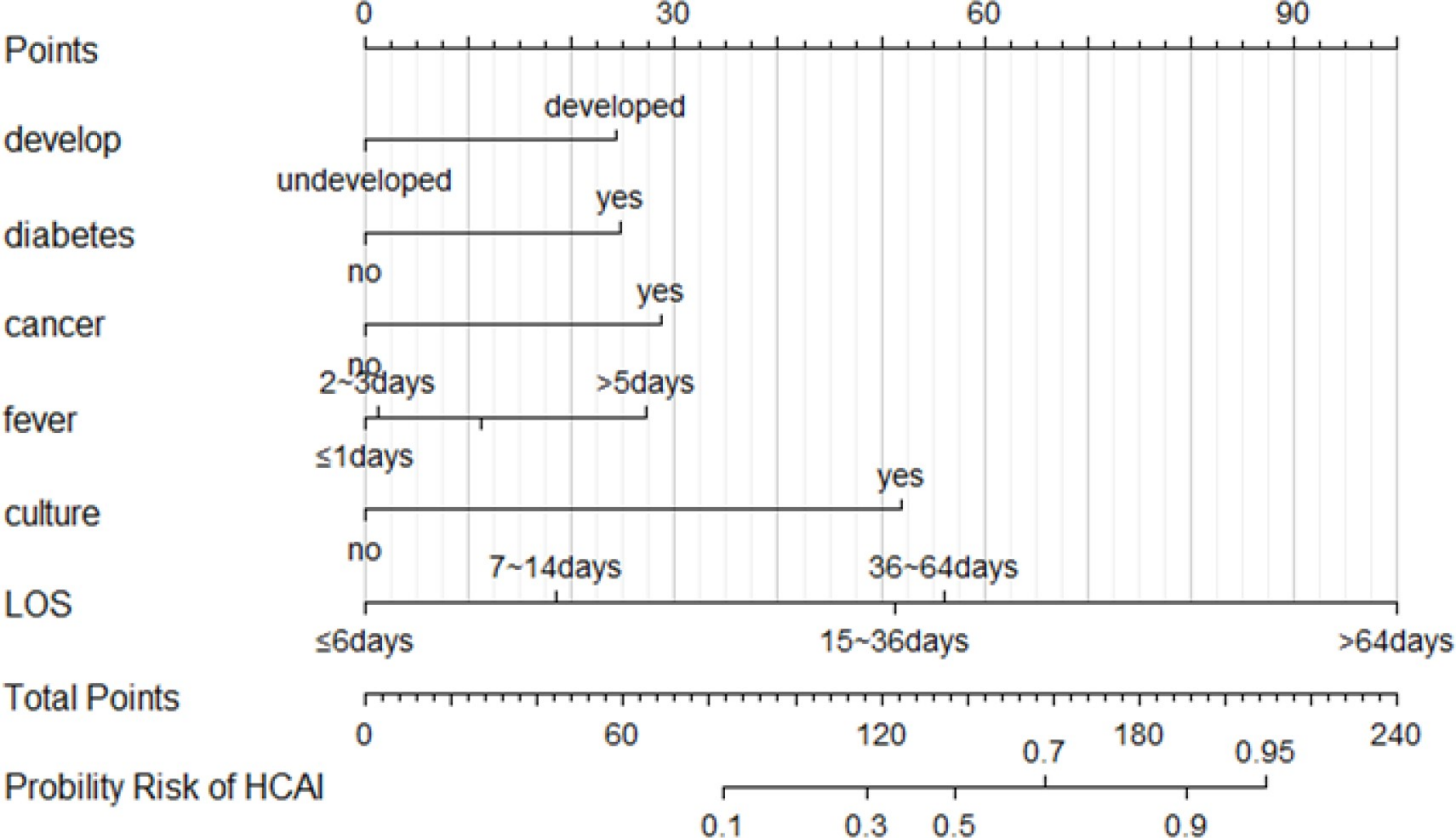
Nomogram of risk factors for the intensive care unit inpatients. To use the nomogram, an individual patient’s value is located on each variable axis, and a line is drawn upward to determine the number of points received for each variable value. The sum of these numbers is located on the total point’s axis, and a line is drawn downward to the probability axes to determine the probability of HCAI. (HCAI) = -5.60+2.11*(Culture = 1/0)+1.01*(Diabetes = 1/0)+1.17*(Cancer = 1/0)+1.00*(Develop = 1/0)+0.75 *(LOS = 7~14days)+2.09*(LOS = 15~36days)+2.29*(LOS = 36~64days)+4.08*(LOS = >64days)+0.05*(Fever = 2~3days)+0.46*(Fever = 4~5days)+1.11* (Fever = >5days). Develop[local economic development levels] (In 2017, real gross domestic product per capita (yuan) is defined as developed, less than 35,000 is defined as underdeveloped);HCAI, healthcare associated infections; LOS, days of hospital stay; fever, persistent fever days; culture, the need for bacterial culture.

The nomograms for predicting HCAI in the ICU patients is shown in Fig 3. The nomogram for predicting HCAI was created based on the following six independent factors identified in the HCAI multiple regression model: local economic development levels (developed or underdeveloped), diabetes (yes or no), cancer (yes or no), persistent fever (≤ 1 day, 2–3 days, 4–5 days or > 5 days), the need for bacterial culture (yes or no) and LOS (≤ 6 days, 7–14 days, 15–36 days, 36–64 days or > 64 days). High scores based on the sum of the assigned number of points for each factor in the nomograms were associated with a high HCAI probability. For example, a patient with an address in an undeveloped region, diabetes, a LOS ≤ 6 days in the past six months, and a persistent fever (> 5 days), without cancer, and requiring bacterial culture would have a total of 132 points (0 points for developmental status, 25 points for diabetes, 28 points for cancer, 27 points for persistent fever, 52 points for bacterial culture and 0 points for a LOS ≤ 6 days). According to the nomogram model, the predicted incidence of HCAI in this patient can be easily observed to be approximately 30%.

### Clinical interactive application

**Fig 4:**
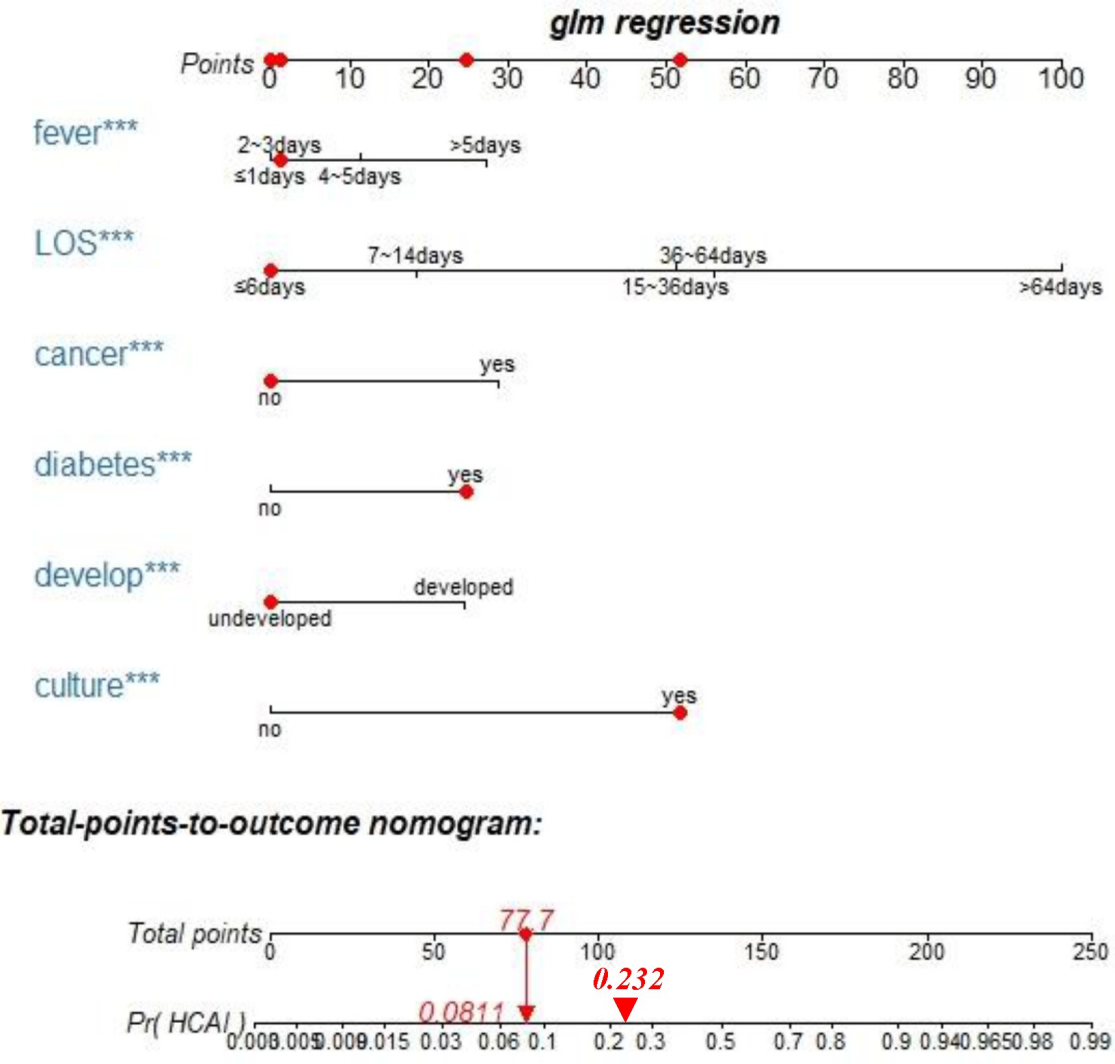
Clinical interactive application. The red points on the six axes of fever, LOS, cancer, diabetes, develop and culture represent individual patient independent variable scores, which are selected by the physician based on the initial consultation. Total points and (Pr) HCAI is the result of the model's automatic display. The red dot on the total points represents the total score of HCAI in the individual patient, and the downward red arrow indicates the probability of the specific HCAI corresponding to the score. The red triangle on the (Pr) HCAI axis represents the cut-off point for HCAI high and low risk based on the ROC cutoff. The right side of 0.232 corresponds to high-risk patients. Develop[local economic development levels] (In 2017, real gross domestic product per capita (yuan) is defined as developed, less than 35,000 is defined as underdeveloped); HCAI, healthcare associated infections; LOS, days of hospital stay; fever, persistent fever days; culture, the need for bacterial culture.

Using the interactive package regplot, healthcare workers can directly click on any position of the independent variable, and based on the nomogram model, the HCAI total score and the specific probability of occurrence will be shown in a direct and very user-friendly manner. For example, a patient with an address in an undeveloped region, diabetes, a persistent fever for 2–3 days, and a LOS ≤ 6 days in the past six months, without cancer, and requiring bacterial culture would have an HCAI total score of 77.7, and the probability of occurrence of 8.11% would be displayed immediately. (Fig 4).

### Outcomes of patients with HCAIs in the ICU

Finally, the main features of HCAI are shown in Table 3. Among the 410 HCAI episodes, the most common infection was lower respiratory tract infection (46.10%), followed by VAP (30.24%). The most common microorganism was *Acinetobacter baumannii* (AB) (43.15%). Multidrug-resistant (MDR) bacteria accounted for 65.23% of all organisms (MDRO/total microorganisms), and the most common MDR bacterium was carbapenem-resistant AB (CR-AB; 23.35%). HCAIs were most likely to occur in winter and spring (62.92%).

**Table 3:** Characteristics of ICU inpatients with HCAI.

Characteristic	Group	Total	χ^2^	P
HCAI	*sum*	410(100.00)		
season	*winter*	180(43.9)	104.21	0.001
	*spring*	78(19.02)		
	*autumn*	76(18.54)		
	*summer*	76(18.54)		
site	*LRT*	189(46.10)	708.49	0.001
	*VAP*	124(30.24)		
	*other*	24(5.85)		
	*bacteremia*	18(4.39)		
	*SSI*	16(3.90)		
	*UTI*	15(3.66)		
	*CAUTI*	15(3.66)		
	*GI*	9(2.20)		
department	*ICU*	308(75.12)	207.01	0.001
	*Non-ICU*	102(24.88)		
microorganisms	*acinetobacter baumannii*	170(43.15)	253.81	0.001
	*other*	64(16.24)		
	*klebsiella pneumoniae*	57(14.47)		
	*staphylococcus aureus*	40(10.15)		
	*pseudomonas aeruginosa*	36(9.14)		
	*escherichia coli*	27(6.85)		
MDRO	*CR-AB*	60(23.35)	39.35	0.001
	*PDR-AB*	52(20.23)		
	*MDR-AB*	40(15.56)		
	*CRE*	31(12.06)		
	*MRSA*	31(12.06)		
	*other*	22(8.56)		
	*MDR-PA*	21(8.17)		

LTR, lower respiratory tract infection; VAP, ventilator-associated pneumonia; SSI: surgical site infection; UTI, urinary Tract Infection; CAUTI, catheter-associated urinary Tract Infection; GI, Gastrointestinal infection; MDRO, multiple drug resistant organism; CR-AB, carbapenem-resistant acinetobacter baumannii; MDR-AB, multidrug resistant acinetobacter baumannii; PDR-AB, pan-drug resistant acinetobacter baumannii; CRE, carbapenem-resistant enterobacteriaceae; MDR-PA, multidrug resistant pseudomonas aeruginosa; MRSA, methicillin-resistant staphylococcus aureus. HCAI, healthcare associated infections.

## Discussion

Most ICU patients are elderly, have severe underlying diseases [24], and are hospitalized acutely for fever caused by severe injuries [25, 26]. In addition, ICU patients are mostly comatose, and invasive surgery and prophylactic antibiotics are routinely used [3, 27]. Furthermore, ICU patients will undergo more clinical procedures, thus increasing the likelihood of cross-infection [28–30]. Therefore, patients in the ICU are at a high risk of HCAIs. Implementing interventions for all risk factors for all patients in the ICU will result in a substantial waste of medical staff labor and medical resources. The nomogram prediction model can identify high-risk patients and independent risk factors, reducing the incidence of HCAI. The method used to create the nomogram has been shown to be reasonable and feasible in several disease models [31–33].

Traditional HCAI risk factor analysis has many shortcomings, and most relevant studies are found to be deficient. In fact, some patients with HCAI during hospitalization are diagnosed after discharge or fail to receive proactive HCAI preventive measures, and the actual clinical application of the abovementioned models has been found to be unsatisfactory. Most relevant studies have only identified a few risk factors that lead to HCAI, but no model has been constructed to predict the probability of HCAI in individual patients. Therefore, we developed a simple, fast and accurate predictive model to proactively prevent the occurrence of HCAIs in ICU patients. When assessing our model, first, no outliers were found, no collinearity was observed, the model showed a good fit with excellent discrimination; and the AUC for the model was greater than 0.84. Second, our nomogram based on the multivariable logistic regression model offers the same diagnostic advantages as the multivariable model. Third, based on the cutoff value of the risk score derived from the training set ROC curve for high- and low-risk classification, the high-risk group had a significantly higher probability of HCAIs. Finally, the model features an easy-to-use interactive tool for healthcare workers.

An interactive nomogram was created using the Rstudio software regplot package. For first-time inpatients with a history of hospitalization (patients with a previous hospitalization in a non-Xinglin hospital), healthcare workers must collect reliable details of the prior admission by depending on a patient’s or family members’ recollection to construct the nomogram. For non-first-time hospitalized patients (the Xinglin system has previous hospitalization information), healthcare workers can directly extract information pertaining to the six independent risk factors using a patient’s hospitalization ID. Patients without a previous admission at any hospital were not included in this analysis. Constructing the interactive nomogram panel requires two simple steps. First, S1 Dataset is downloaded and stored locally, and second, all codes (S1 File) are run in Rstudio software. Then healthcare workers can dynamically visualize individual patients’ HCAI probabilities and specific clinical options by clicking on the relevant features of the user interface. When the score falls to the left side of the 0.232 cutoff point for high and low HCAI risk, a low-risk HCAI prevention strategy can be used, thus maximizing limited resources. In addition, we built a simple HCAI calculator using Excel and directly obtained the risk ratio of HCAI by selecting the characteristics of different individuals (S2 Dataset).

The need for bacterial culture was an independent risk factor for HCAI, suggesting that healthcare workers played an important role in the prediction of HCAI. Bacterial culturing is required for a clinical decision made by healthcare workers after synthesizing the basic information from the patient's last hospitalization. During the same hospitalization, bacterial culturing is required, which means that the risk of CAI increases. The corresponding probability of CAI increases, which further leads to the occurrence of a high risk of HCAI [11, 34]. For high-HCAI risk patients, the hospital infection management department should take the proper initiatives to prevent HCAIs, requiring clinical healthcare workers to devote more time and energy, taking the greatest degree of care. Therefore, there is still an opportunity to prevent a subsequent HCAI. Russo et al. [35] and Schroder et al. [36] found similar results.

One of the most interesting findings of this study was that economic development in the region in which a patient lived was an independent risk factor for HCAI. Patients from developed regions had a higher probability of developing an HCAI than patients from regions with a lower per capita GDP. The possible reasons are as follows: (1) The “siphon effect” in developed areas is distinct, with a high medical technology level and a low missed diagnosis rate. (2) Hospitals in underdeveloped areas cannot treat patients with complicated conditions (for example, late-stage HCAI patients), resulting in the transfer of such patients to higher-level medical institutions. (3) Patients in developed areas have greater economic means and greater medical literacy than those in developing areas. Most patients with severe illness will directly select the best medical care in the region.

Our study further found that MDR organisms were the main cause of HCAIs, namely, AB, CR-AB, pan-drug-resistant AB (PDR-AB) and multidrug-resistant AB (MDR-AB), together accounting for 35.78% of all organisms, which is consistent with the results of a systematic regression analysis [11] and is greater than the proportions reported in relevant Western literature [37, 38]. LRT infections are the most common HCAIs, followed by VAP, and CAUTI ranks third, which is consistent with the results of most studies [39–41]. From a different perspective, this finding indicates that invasive procedures are common in the ICU and increase the likelihood of device-related HCAIs. In our study, HCAIs were mainly caused by gram-negative pathogens. Viderman et al. and Kolpa et al. also obtained similar results [10, 42]. AB (43.15%) and *Staphylococcus aureus* (10.15%) accounted for the highest proportions of gram-negative and gram-positive bacteria, respectively, which is consistent with the results of Kolpa et al. [42] The winter and spring seasons showed the highest incidence rates of HCAIs. Previous studies have confirmed the effect of seasonal changes on the prevalence of HCAIs [43].

Our study has some limitations. First, as a retrospective study, a certain degree of selection bias may exist. Prospective studies are needed to verify the accuracy of our nomograms, and more rigorous methods are required to illustrate the risk factors of HCAI. Second, obesity could not be fully assessed because of the lack of data used to calculate the body mass index of all patients. Third, we did not have data on smoking, alcohol consumption, diet or physical activity indicators; therefore, the impact of healthy behaviors on HCAIs was not evaluated. Fourth, we lacked external data validation but performed effective internal verification. Fifth, this model only applies to those patients who have a history of previous hospitalization. Sixth, it would have been helpful to calculate the time between the bacterial culturing and HCAI diagnosis, but such data could not be attained. Finally, the six hospitals examined were the only pilot hospitals in Guizhou Province that had implemented professional hospital infection surveillance software and were limited to tertiary and secondary general hospitals. The institutions do not represent all Guizhou hospitals and specialist hospitals; however, the sample population covered all nine cities and prefectures in the province and was widely distributed.

In conclusion, the main features of HCAI were as follows: the most common site of infection was the LRT. AB was the most frequently detected bacterium. MDR bacteria were mainly gram-negative bacteria such as CR-AB. Most HCAIs occurred in winter and spring. Our results demonstrated that six independent risk factors were associated with HCAI in the ICU, namely, the developmental status of the region, the need for bacterial cultures, a history of diabetes or cancer, a long LOS and persistent fever. The interactive nomogram model can be widely adopted in a wide range of clinical settings. The model can quickly and objectively visualize the extent of the HCAI risk in individual patients and identify high-risk patients to facilitate adjustments to nursing care strategies. Prospective studies should be conducted to improve the model.

## Supporting information

S1 Table Comparison of major differences of healthcare-associated infection diagnosis criteria issued by National Health and Family Planning Commission of the People’s Republic of China (NHFPC; formerly the Chinese Ministry of Health) and US Centers for Disease Control and Prevention (CDC)/National Healthcare Safety Network (NHSN). (PDF)

S1 Fig Model outlier diagnosis. (TIF)

S1 File The code of interactive nomograms. (PDF)

S1 Dataset (CSV)

S2 Dataset HCAI calculator from the regplot model. (XLSX)
