# Supplementary material for: An interactive nomogram to predict healthcare-associated infections in ICU patients: A multicenter study in GuiZhou Province, China
Source: PLoS One. 2019 Jul 15;14(7):e0219456. doi: 10.1371/journal.pone.0219456 (PMC6629073; doi:10.1371/journal.pone.0219456)
Supplement: S1 Table — (PDF) [file pone.0219456.s001.pdf]

S1 Table. Comparison of major differences of healthcare-associated infection diagnosis criteria issued by National Health and Family Planning Commission of the People's Republic of China (NHFPC; formerly the Chinese Ministry of Health) and US Centers for Disease Control and Prevention (CDC)/National Healthcare Safety Network (NHSN).

Items	NHFPC definition	CDC/NHSN definition
Year of publication	2001, not updated	1987, updated nearly every year
Major types of infection	· Bone and joint infection<br>· Bloodstream infection (BSI)<br>· Central nervous system infection<br>· Cardiovascular system infection<br>· Eye, ear, nose, throat, or mouth infection<br>· Gastrointestinal system infection<br>· Lower respiratory tract infection (pneumonia included)<br>· Reproductive tract infection<br>· Skin and soft tissue infection<br>· Surgical site infection (SSI)	· Bone and joint infection<br>· Bloodstream infection<br>· Central nervous system infection<br>· Cardiovascular system infection<br>· Eye, ear, nose, throat, or mouth infection<br>· Gastrointestinal system infection<br>· Pneumonia (PNEU) infections<br>· Lower respiratory system infection, other than pneumonia<br>· Reproductive tract infection<br>· Skin and soft tissue infection<br>· SSI<br>· UTI

	· Upper respiratory tract infection<br>· Urinary tract infection (UTI)	
Device-associated infections	Not defined	Cleary defined
Clostridium difficile infection	Not defined	Cleary defined as one important gastrointestinal system infection
Infection for patient ≤1 year of age	Not specially mentioned excepted for cardiovascular and central nervous system infections	Specially defined in many other infections, including urinary system infection, pneumonia, bloodstream infection
Classification of an infection as present on admission (POA) or a healthcare-associated infection (HCAI)	HCAI primary refers to infection acquired >48 h after admission, unless there is a clear incubation period for the infection	Many objective surveillance definitions and guidance were used, e.g. 7-day infection window period, date of event, POA, HCAI, 14-day repeat infection timeframe (RIT), secondary bloodstream infection attribution period, pathogen assignment guidance

Ventilator-associated pneumonia (VAP) was defined in patients who had pneumoconiosis 2 calendar days or later after mechanical ventilation on the day the event occurred, referred to as ventilator placement day 1; catheter-associated urinary tract infection (CAUTI) was defined as a urinary tract infection which presented more than 2 calendar days after placement of an indwelling catheter, referred to as placement day 1; central venous catheter-related bloodstream infection (CLABSI) was defined as laboratory-confirmed bloodstream infection when the centerline or umbilical cord catheter was present for more than 2 calendar days after the date of the event, referred to as equipment placement day 1.
