# Supplementary figures and images for: An interactive nomogram to predict healthcare-associated infections in ICU patients: A multicenter study in GuiZhou Province, China
Source: PLoS One. 2019 Jul 15;14(7):e0219456. doi: 10.1371/journal.pone.0219456 (PMC6629073; doi:10.1371/journal.pone.0219456)

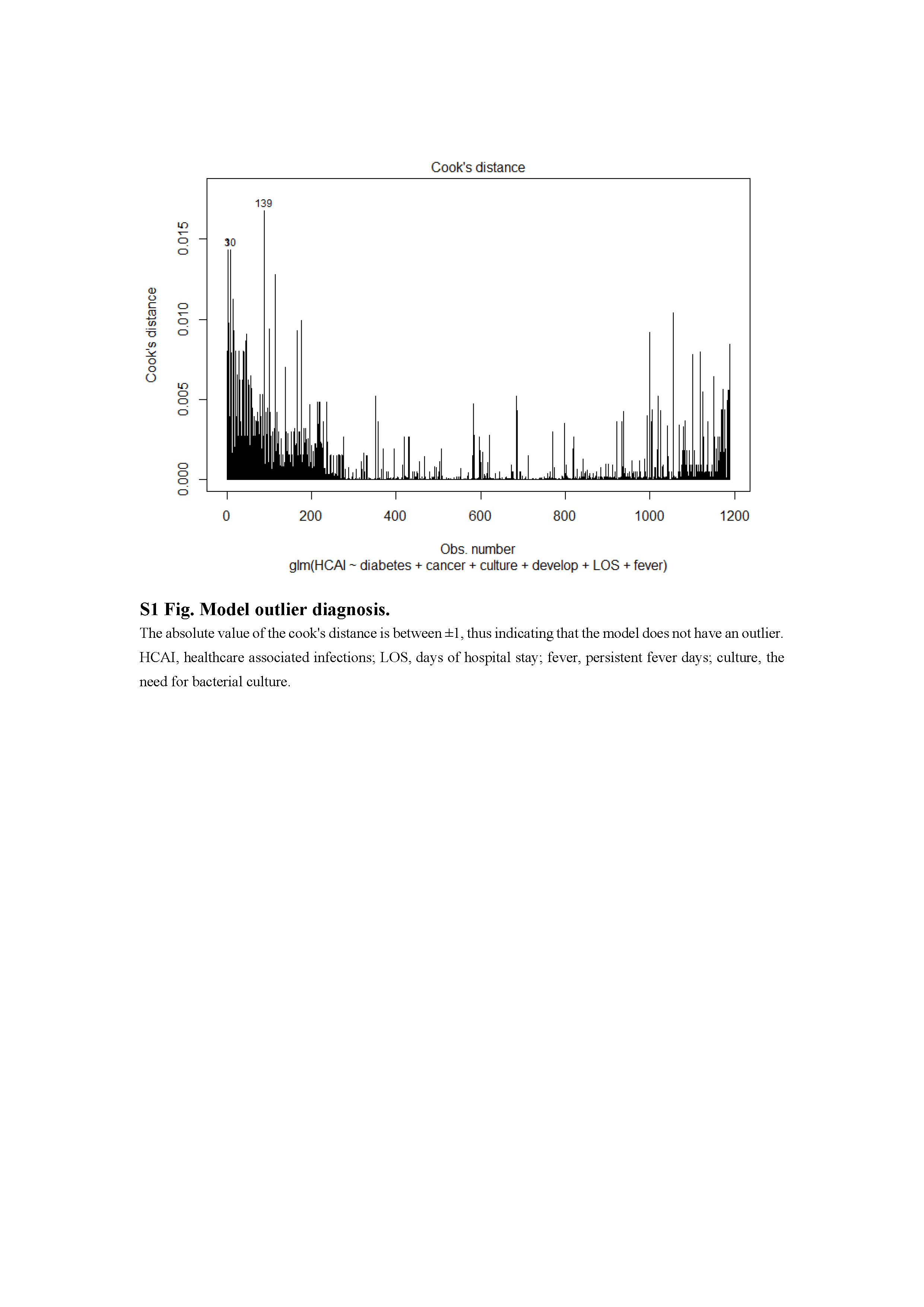

Supplement: S1 Fig — (TIF) [file pone.0219456.s002.tif]
