# Supplementary material for: An interactive nomogram to predict healthcare-associated infections in ICU patients: A multicenter study in GuiZhou Province, China
Source: PLoS One. 2019 Jul 15;14(7):e0219456. doi: 10.1371/journal.pone.0219456 (PMC6629073; doi:10.1371/journal.pone.0219456)
Supplement: S1 File — (PDF) [file pone.0219456.s003.pdf]

S1 The code of interactive nomograms

```
################################Part 1, Load packet
if(!require(dplyr)) install.packages("dplyr")
library(dplyr)
if(!require(stringr)) install.packages("stringr")
library(stringr)
if(!require(tidyverse)) install.packages("tidyverse")
library(tidyverse)
if(!require(foreign)) install.packages("foreign")
library(foreign)
if(!require(regplot)) install.packages("regplot")
library(regplot)

################################Part 2, Set file path(Path is where the data is stored on the computer)
setwd("D:\\R work")

################################Part 3, Read data set
ICU <- read.csv("D:\\R work\\ICU.csv")

################################Part 4, Extract training set and validation set
dev <- ICU[ICU$group == "train", ]
vad <- ICU[ICU$group == "validation", ]

############################Part 5, Reset reference standard
levels(dev$culture)
levels(dev$cancer)
levels(dev$diabetes)
levels(dev$hypertension)
dev$develop <- relevel(dev$develop, "undeveloped")
levels(dev$develop) <- list("undeveloped" = 0, "developed" = 1)
dev$LOS <- relevel(dev$LOS, "≤6days")
levels(dev$LOS) <- list("≤6days" = 0, "7~14days" = 1, "15~36days" = 2, "36~64days" = 3, ">64days" = 4)
dev$fever <- relevel(dev$fever, "≤1days")
levels(dev$fever) <- list("≤1days" = 0, "2~3days" = 1, "4~5days" = 2, ">5days" = 3)
dev$antsD <- relevel(dev$antsD, "≤3days")
levels(dev$antsD) <- list("≤3days" = 0, "4~6days" = 1, "7~12days" = 2, "13~17days" = 3, "18~37days" = 4, ">37days" = 5)

########################Part 6， construct and drawing interactive nomograms for clinical applications
modelc <- glm(HCAI ~ culture + develop + diabetes + cancer + LOS + fever, data = dev, family = binomial(link = "logit"))
```

```
summary(modelc)
round(cbind(coef=coef(modelc), confint.default(modelc)),2)
round(exp(cbind(OR = coef(modelc), confint(modelc))),2)

dev$predmodelc <- predict(newdata = dev, modelc, "response")
regplot(modelc, observation = dev[3,])
```
